# The extended finite element method in endodontics: A scoping review and future directions for cyclic fatigue testing of nickel–titanium instruments

**DOI:** 10.1002/cre2.893

**Published:** 2024-05-21

**Authors:** Philip Yuan‐Ho Chien, Laurence James Walsh, Ove Andreas Peters

**Affiliations:** ^1^ The University of Queensland, School of Dentistry, UQ Oral Health Centre Herston QLD Australia

**Keywords:** finite element analysis, nitinol, root canal

## Abstract

**Objectives:**

The present study reviews the current literature regarding the utilization of the extended finite element method (XFEM) in clinical and experimental endodontic studies and the suitability of XFEM in the assessment of cyclic fatigue in rotary endodontic nickel–titanium (NiTi) instruments.

**Material and Methods:**

An electronic literature search was conducted using the appropriate search terms, and the titles and abstracts were screened for relevance. The search yielded 13 hits after duplicates were removed, and four studies met the inclusion criteria for review.

**Results:**

No studies to date have utilized XFEM to study cyclic fatigue or crack propagation in rotary endodontic NiTi instruments. Challenges such as modelling material inputs and fatigue criteria could explain the lack of utilization of XFEM in the analysis of mechanical behavior in NiTi instruments.

**Conclusions:**

The review showed that XFEM was seldom employed in endodontic literature. Recent work suggests potential promise in using XFEM for modelling NiTi structures.

## 1 INTRODUCTION

Fracture of engine‐driven rotary nickel–titanium (NiTi) endodontic instruments can occur via cyclic fatigue; a phenomenon by which cracks propagate due to the repeated tensile‐compressive while the instrument rotates freely in a curved canal (Chien et al., 2023; Pedullà et al., 2018; Pruett et al., 1997). Instrument fracture during endodontic treatment is of concern to the practicing clinician as such events could impact the prognosis of treatment should the instrument fragment compromise chemomechanical cleansing, working length control, and root canal filling (McGuigan et al., 2013; Sjogren et al., 1990).

Numerous studies on the cyclic fatigue of rotary NiTi instruments used in endodontic literature have investigated either variations to the study design or the type of proprietary rotary instrument used (Hülsmann et al., 2019). Study designs for cyclic fatigue can be classified as static or dynamic; however, there appears to be no consensus as to the best approach for analyzing cyclic fatigue, which brings into question the clinical relevance of these experiments (Hülsmann et al., 2019).

Recently, finite element analysis (FEA) has been explored as a tool to support and validate experimental findings from benchtop cyclic fatigue testing (Chien et al., 2021). FEA is a numerical analysis where a physical model is subdivided into smaller “finite” elements to form a mesh model, which can then be investigated via computational algorithms to establish the relationships between these elements (Chien et al., 2021). Multiple studies have used FEA to visualize stresses within rotary NiTi instruments during the shaping of the root canal and to provide insight into their mechanical behavior (Arbab‐Chirani et al., 2011; Chien et al., 2023; Ha et al., 2015; Kim et al., 2008, 2009; Lee et al., 2011).

However, FEA is limited in the assessment of crack propagation, which is the process that ultimately leads to fracture (separation) of rotary instruments (Chien et al., 2023). Essentially, a crack is a geometric discontinuity of a material, and fracture mechanics is the study of the evolution of the discontinuity (Recho, 2012). Cracks have complicated geometries with arbitrary propagation paths, and unsurprisingly this poses a challenge in fracture mechanics where propagation occurs on curved or kinked paths within three‐dimensional structures (Zhuang et al., 2014). Internal defects such as material interfaces, cracks, voids, and inclusions create difficulty in the meshing process for FEA (Zhuang et al., 2014). As finite element (FE) methods require piecewise differentiable polynomial approximations, they are unsuitable for problems with discontinuous solutions (Yazid et al., 2009). In the case of modelling crack propagation, the problem involves evolving discontinuities that must be regenerated at each step (Yazid et al., 2009). Furthermore, FEA only allows cracks to propagate along the element edge and not along a natural arbitrary path (Zhuang et al., 2014).

Accordingly, improvements have since been made to FEA to overcome these limitations. The extended finite element method (XFEM) is a numerical method used to model both internal and external boundaries (e.g., holes, inclusions, and cracks) without requiring the mesh to conform to these boundaries (Yazid et al., 2009). XFEM has a significant improvement in crack modeling with the development of a partition of unity‐based enrichment method for discontinuous fields (Khoei, 2014). XFEM allows for the automatic insertion and extension of cracks inside an element, and it can model propagation from element to element (Boonrawd et al., 2022). Naturally, these advantages could be useful in the modelling of fracture events in NiTi endodontic instruments.

This study explores the current utilization of XFEM in the endodontic literature and assesses its suitability for modelling of crack propagation in NiTi endodontic instruments.

## 2 LITERATURE SEARCH STRATEGY

A literature search was conducted in November 2023 in the PubMed and Scopus database using the following search terms (extended finite element method[All fields] OR XFEM[All fields]) AND (endodontic[All fields] OR nickel‐titanium[All fields] OR endodontic instrument[All fields] OR root canal[All fields] OR pulp chamber[All fields]). An adapted PRISMA (Preferred Reporting Items for Systematic Reviews and Meta‐Analyses) literature search strategy was employed to identify suitable papers for review (Moher et al., 2009). The language of publication was restricted to English only. The inclusion criteria required studies that used XFEM in both clinical and experimental studies in the field of endodontics where the root canal system was involved. Conference abstracts were excluded from the search, and no previous reviews on this topic were identified.

## 3 RESULTS

**Figure 1:**
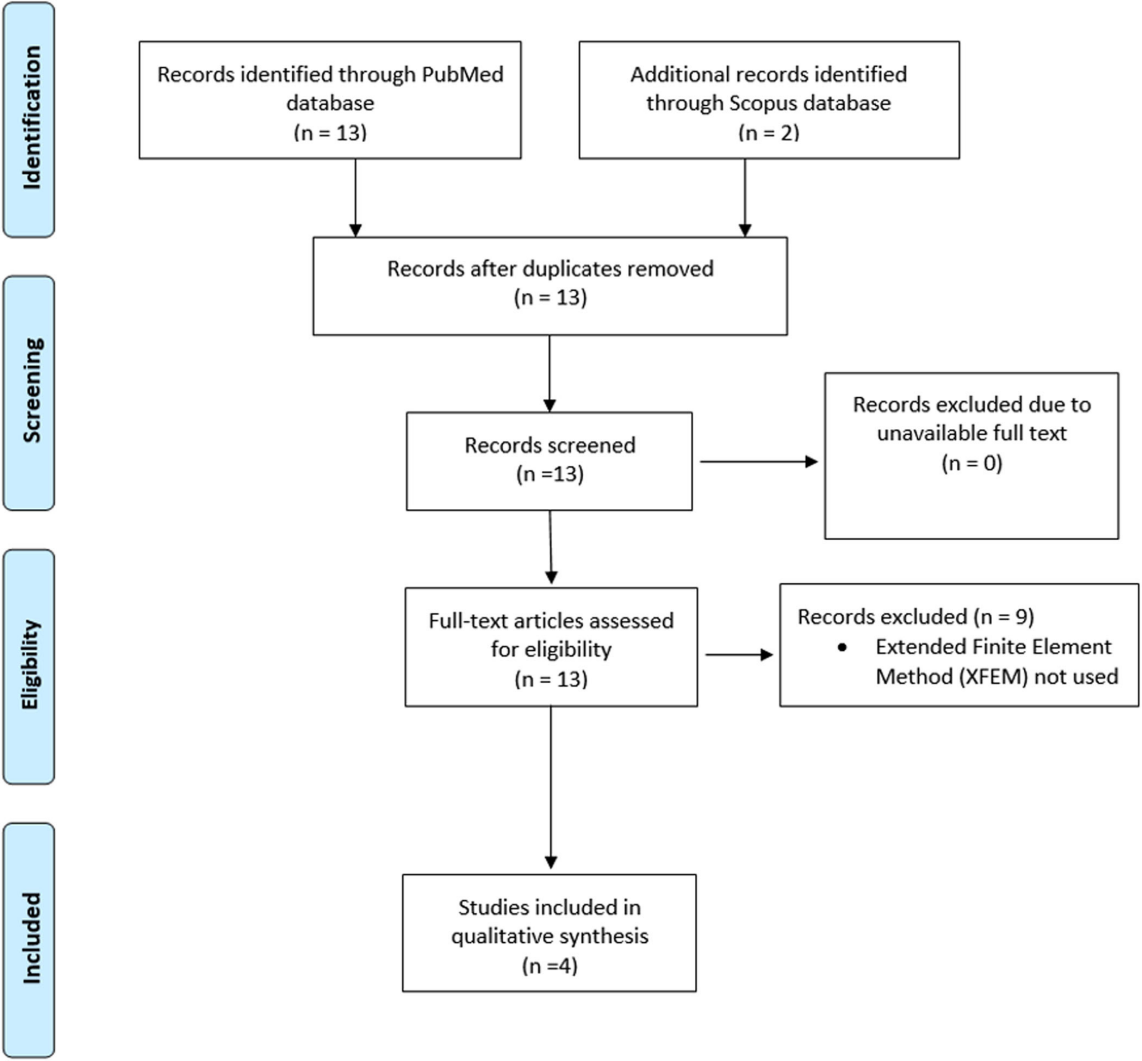
Adapted PRISMA (Preferred Reporting Items for Systematic Reviews and Meta‐Analyses) flow diagram detailing the literature search strategy (Moher et al., 2009).

The search yielded 15 hits in total between PubMed and Scopus databases, of which two were duplicates and were subsequently excluded. The 13 titles and abstracts of available English articles were screened for relevance, and all met the inclusion criteria. The 13 full‐text articles were then assessed for eligibility, and a further nine articles were excluded as XFEM was not employed in the methodology. No further studies were identified using cited reference searching or snowballing (Streeton et al., 2004). In total, four articles were selected for the current review (Figure 1).

In all four selected studies, endodontically treated teeth were the subject of examination. XFEM was employed to analyze stress distribution and crack propagation in enamel (Liu et al., 2021), dentine (Boonrawd et al., 2022; Liu et al., 2021; Zhang et al. 2015, 2019), and in restorative (material) interfaces such as in glass ionomer cement and adhesive resin layers (Zhang et al., 2019). None of the studies used XFEM to analyze endodontic instruments. A summary of the findings in each study is detailed in Table 1.

**Table 1:** Summary of FE models and XFEM utilization in selected studies.

Study	FE modelling	XFEM utilization
Zhang et al. (2015)	Modelled six endodontically treated, maxillary central incisors and decoronated with varying combinations of labial and palatal ferrule (either 0, 1, or 2 mm). A post, standardized core and a full coverage metal crown were used to restore the coronal portion of the incisors	Analyzed stress distribution and crack propagation in cervical dentine
	A static load of 350 N was applied to the lingual surface, 2 mm below the incisal edge of the crown	
Zhang et al. (2019)	Modelled a natural tooth and three endodontically treated maxillary first molars. Models varied by the type of access cavity preparations Four static loads of 200 N (800 N) total were applied on contact points registered in centric occlusion	Analyzed stress distribution and crack propagation in the glass ionomer cement layer, adhesive resin cement layer, and root dentine
Liu et al. (2021)	Modelled an endodontically treated mandibular first premolar with an h‐shaped canal. Using an identical tooth model, four models were generated with varying access cavity designs (intact, conservative, modified‐conservative, and traditional) A static load of 600 N (300 N on the buccal cusp and buccal side of the distal fossa) was applied	Analyzed stress distribution and crack propagation in enamel and dentine
Boonrawd et al. (2022)	Modelled four different endodontically treated mandibular first molars with full coverage zirconia crowns. Models varied by their periodontal pocket depth, crack level, and extension of composite resin core A static load of 700 N was applied at eight loading points on the occlusal surface	Analyzed stress distribution and crack propagation in dentine

Abbreviations: FE, finite element; XFEM, extended finite element method.

## 4 DISCUSSION

The fatigue failure process can be divided into three stages, which are:

1. The formation of cracks (crack initiation).
2. Crack propagation (or crack growth).
3. Fracture to failure (Zhou et al., 2022).

To date, no studies have used XFEM to investigate crack phenomena in endodontic NiTi instruments. From the four studies selected, XFEM has been utilized for assessing crack propagation, but in the context of tooth survivability. All identified studies varied in the study design used to assess the impact of access cavities, ferrules, or restorative considerations on the likelihood of crack formation in the presence of a static load that is likely to be found in normal occlusal function. However, the strengths and limitations of the reviewed studies can provide insight into the suitability of XFEM for the study of cyclic fatigue failure in NiTi instruments.

In the 2015 study of Zhang et al., the numerical predictions and experimental results were in good agreement with regard to ultimate fracture failure and the fracture locations for maxillary central incisors under static load (Zhang et al., 2015). XFEM analysis clarified susceptible areas for crack formation in endodontically treated teeth and gave possible models for fracture behavior. However, the authors noted that the cracking model could not account for fatigue‐induced failures from the conventional static loading of these teeth, and followed static fracture mechanics (Zhang et al., 2015). As fractures in prosthodontic restorations occur due to cyclic loading rather than a singular acute overload, static fracture mechanics may not be an accurate representation of clinical reality (Valdivia et al., 2012; Zhang et al., 2015). The same could be said about NiTi endodontic instruments used during shaping and cleaning of root canals: instrument fracture does not always occur due to loads that exceed the elastic limit of the instruments, but in situations where the instrument is allowed to rotate freely in a canal (Pruett et al., 1997). Future study designs should consider dynamic loads over static loads to mimic clinical reality. Previous work in other biomedical applications has modelled cyclic loading in XFEM studies, such as mandibular reconstruction plates (Wan et al., 2021) and metal fiber‐reinforced polymer composites (Rashnooie et al., 2023). Such concepts could be brought into the realm of loading conditions on NiTi instruments.

Studies using conventional FEA have already been able to predict fracture locations of NiTi instruments in cyclic action to a reasonable degree of accuracy (Chien et al., 2023; Scattina et al., 2015). However, without a fatigue criterion or fatigue model, the prediction of fatigue life would still be limited using either FEA or XFEM techniques in endodontic instruments. In other words, future research could focus on the first stage of the fatigue process (crack initiation) rather than the postmortem approach of locating the region of failure. In a previous study, the fatigue phenomenon of a mandibular reconstruction plate has already been modelled with a two‐phase crack initiation (by accounting for the correct material constants and the hysteresis strain energy per cycle) and propagation process (via a crack evolution model through application of the Paris' law) (Wan et al., 2022). Other studies have also suggested various equations for crack modelling (Shahmoradi et al., 2022; Tian et al., 2019). Logically, applying similar concepts for a rotary instrument inside a canal should theoretically be no different provided the correct material constants are considered for a similar two‐phase crack modelling process.

Three of the four included studies assumed the materials investigated to be either brittle or quasi‐brittle (Liu et al., 2021; Zhang et al. 2015, 2019). Brittle materials have extremely low plasticity and ductility, and cracks can initiate without plastic deformation, leading to brittle breakage (Huang et al., 2018). On the other hand, NiTi alloys exhibit excellent ductility (Yahia et al., 2009) and thus XFEM alone may not be suitable for modelling cracks in ductile materials, given that a significant plastic region occurs before tensile failure (Kumar & Bhardwaj, 2018). To complicate matters, NiTi alloys do not exhibit linear elastic deformation, due to the formations of intermediate transformation states such as the rhombohedrally distorted phase (R‐phase) when load is applied (Chien et al., 2022). Previous work has demonstrated that these regions of plastic deformation are seen before fracture (Chien et al., 2022).

In both traditional FEA and XFEM investigations, the analyses are only as accurate as the material inputs given. In all studies reviewed, mechanical properties such as Young's modulus, Poisson ratio, tensile strength, and fracture toughness of the investigated tissues and materials (such as enamel, dentin, pulp, resin, glass ionomer cement, gutta‐percha, periodontal ligament, alveolar bone) were provided using experimental data sourced from previous studies. NiTi alloys used in endodontic instruments do not have readily available experimental data for mechanical inputs, as most data on these alloys is considered proprietary information or trade secrets (Zupanc et al., 2018). Many NiTi endodontic instruments in the current market have undergone various thermomechanical treatments to enhance their performance during the chemomechanical preparation of root canals. Postmanufacture processing of NiTi alloys such as Blue and Gold treatment may not necessarily alter the core composition of NiTi, but it can alter the thresholds for martensitic and austenitic transformations (Chien et al., 2022). Given that there exist over 160 proprietary endodontic instruments in the global market (Gavini et al., 2018), it may be difficult to model accurately each instrument without access to all of their individual mechanical parameters.

Despite these challenges, studies continue to improve and refine XFEM techniques to provide more accurate modelling. Additional enrichment functions, such as those detailed by Kumar and Bhardwaj in 2018, may need to be employed to account for the ductile nature of NiTi alloys when testing (Kumar & Bhardwaj, 2018). Zhang et al. in 2022 noted that crack initiation and propagation behaviors in NiTi shape memory alloy (SMA) had yet to be reported, and they were the first to model crack propagation in NiTi SMA‐containing amorphous zones and phases (Zhang et al., 2022). The cohesive model is also a potential approach which has been successfully used in previous analyses of ductile crack propagation problems in metallic materials (Li et al., 2021). Such work could be translated in the context of NiTi instrument models and their cyclic behavior in a root canal, but could still be difficult due to the complexity of the physical model, material properties and rotational movement. The limitations of this study are that there were no prior works identified that employed XFEM specifically on NiTi instruments, and consequently only related studies were available for qualitative synthesis. The suggested recommendations for future studies are therefore speculative in nature.

## 5 CONCLUSIONS

The present review revealed that XFEM has only been employed in four studies in endodontic literature that included modelling of the root canal system. Of the studies identified, XFEM usage was focused on the analysis of crack propagation for teeth and tooth models, and no studies had yet used XFEM to analyze crack propagation in NiTi alloy endodontic instruments. Modelling cyclic fatigue of rotary NiTi instruments using XFEM could prove challenging due to the complex material properties of NiTi, and this approach may be limited for the prediction of fatigue life without a failure criterion. Nevertheless, recent work shows potential promise in using XFEM for modelling NiTi structures, particularly in understanding crack propagation.

## AUTHOR CONTRIBUTIONS

**Philip Yuan‐Ho Chien, Laurence James Walsh, and Ove Andreas Peters**: Conceptualization. **Philip Yuan‐Ho Chien**: Methodology; formal analysis; investigation; data curation; writing—original draft preparation; visualization. **Laurence James Walsh and Ove Andreas Peters**: Validation; writing—review and editing; supervision; project administration. **Laurence James Walsh and Ove Andreas Peters**: Validation; writing—review and editing; supervision; project administration.

## CONFLICT OF INTEREST STATEMENT

The authors declare no conflict of interest.

## Data Availability

Data sharing is not applicable to this article as no data sets were generated or analyzed during the current study.

## References

[cre2893-bib-0001] Arbab‐Chirani, R. , Chevalier, V. , Arbab‐Chirani, S. , & Calloch, S. (2011). Comparative analysis of torsional and bending behavior through finite‐element models of 5 Ni–Ti endodontic instruments. Oral Surgery, Oral Medicine, Oral Pathology, Oral Radiology, and Endodontology, 111(1), 115–121. 10.1016/j.tripleo.2010.07.017 21176826

[cre2893-bib-0002] Boonrawd, N. , Rungsiyakull, P. , Rungsiyakull, C. , & Louwakul, P. (2022). Effects of composite resin core level and periodontal pocket depth on crack propagation in endodontically treated teeth: An extended finite element method study. The Journal of Prosthetic Dentistry, 128(2), 195.e1–195.e7. 10.1016/j.prosdent.2022.05.003 35779973

[cre2893-bib-0003] Chien, P. Y.‐H. , Martins, J. N. R. , Walsh, L. J. , & Peters, O. A. (2022). Mechanical and metallurgical characterization of nickel–titanium wire types for rotary endodontic instrument manufacture. Materials, 15(23), 8367. 36499875 10.3390/ma15238367 PMC9737817

[cre2893-bib-0004] Chien, P. Y.‐H. , Walsh, L. J. , & Peters, O. A. (2021). Finite element analysis of rotary nickel–titanium endodontic instruments: A critical review of the methodology. European Journal of Oral Sciences, 129(5), e12802. 34105190 10.1111/eos.12802

[cre2893-bib-0005] Chien, P. Y.‐H. , Wan, B. , Walsh, L. J. , & Peters, O. A. (2023). Experimental and 2‐step finite element analysis of cyclic fatigue resistance of conventional and heat‐treated rotary endodontic nickel–titanium instruments. Applied Sciences, 13(4), 2080.

[cre2893-bib-0006] Gavini, G. , Santos, M. , Caldeira, C. L. , Machado, M. E. L. , Freire, L. G. , Iglecias, E. F. , Peters, O. A. , & Candeiro, G. T. M. (2018). Nickel–titanium instruments in endodontics: A concise review of the state of the art. Brazilian Oral Research, 32, 44–65. 10.1590/1807-3107bor-2018.vol32.0067 30365608

[cre2893-bib-0007] Ha, J. H. , Cheung, G. S. P. , Versluis, A. , Lee, C. J. , Kwak, S. W. , & Kim, H. C. (2015). ‘Screw‐in’ tendency of rotary nickel–titanium files due to design geometry. International Endodontic Journal, 48(7), 666–672. 25088359 10.1111/iej.12363

[cre2893-bib-0008] Huang, Z. , Li, G. , Tian, S. , Song, X. , Sheng, M. , & Shah, S. (2018). Chapter One—Theoretical basis of abrasive jet. In Z. Huang , G. Li , S. Tian , X. Song , M. Sheng , & S. Shah (Eds.), Abrasive water jet perforation and multi‐stage fracturing (pp. 1–62). Gulf Professional Publishing.

[cre2893-bib-0009] Hülsmann, M. , Donnermeyer, D. , & Schäfer, E. (2019). A critical appraisal of studies on cyclic fatigue resistance of engine‐driven endodontic instruments. International Endodontic Journal, 52(10), 1427–1445. 31267579 10.1111/iej.13182

[cre2893-bib-0010] Khoei, A. (2014). Introduction. Extended Finite Element Method, 1–29.

[cre2893-bib-0011] Kim, H.‐C. , Cheung, G. S.‐P. , Lee, C.‐J. , Kim, B.‐M. , Park, J.‐K. , & Kang, S.‐I. (2008). Comparison of forces generated during root canal shaping and residual stresses of three nickel–titanium rotary files by using a three‐dimensional finite‐element analysis. Journal of Endodontics, 34(6), 743–747. 18498904 10.1016/j.joen.2008.03.015

[cre2893-bib-0012] Kim, H. C. , Kim, H. J. , Lee, C. J. , Kim, B. M. , Park, J. K. , & Versluis, A. (2009). Mechanical response of nickel–titanium instruments with different cross‐sectional designs during shaping of simulated curved canals. International Endodontic Journal, 42(7), 593–602. 19467053 10.1111/j.1365-2591.2009.01553.x

[cre2893-bib-0013] Kumar, S. , & Bhardwaj, G. (2018). A new enrichment scheme in XFEM to model crack growth behavior in ductile materials. Theoretical and Applied Fracture Mechanics, 96, 296–307.

[cre2893-bib-0014] Lee, M.‐H. , Versluis, A. , Kim, B.‐M. , Lee, C.‐J. , Hur, B. , & Kim, H.‐C. (2011). Correlation between experimental cyclic fatigue resistance and numerical stress analysis for nickel–titanium rotary files. Journal of Endodontics, 37(8), 1152–1157. 21763912 10.1016/j.joen.2011.03.025

[cre2893-bib-0015] Li, H. , Li, L. , Fan, J. , & Yue, Z. (2021). Verification of a cohesive model‐based extended finite element method for ductile crack propagation. Fatigue & Fracture of Engineering Materials & Structures, 44(3), 762–775.

[cre2893-bib-0016] Liu, Y. , Liu, H. , & Fan, B. (2021). Influence of cavity designs on fracture behavior of a mandibular first premolar with a severely curved h‐shaped canal. Journal of Endodontics, 47(6), 1000–1006. 33775730 10.1016/j.joen.2021.03.012

[cre2893-bib-0017] McGuigan, M. B. , Louca, C. , & Duncan, H. F. (2013). Clinical decision‐making after endodontic instrument fracture. British Dental Journal, 214(8), 395–400. 23619858 10.1038/sj.bdj.2013.379

[cre2893-bib-0018] Moher, D. , Liberati, A. , Tetzlaff, J. , & Altman, D. G. (2009). Preferred Reporting Items for Systematic Reviews and Meta‐Analyses: The PRISMA statement. PLoS Medicine, 6(7), e1000097. 19621072 10.1371/journal.pmed.1000097 PMC2707599

[cre2893-bib-0019] Pedullà, E. , Lo Savio, F. , La Rosa, G. R. M. , Miccoli, G. , Bruno, E. , Rapisarda, S. , Chang, S. W. , Rapisarda, E. , La Rosa, G. , Gambarini, G. , & Testarelli, L. (2018). Cyclic fatigue resistance, torsional resistance, and metallurgical characteristics of M3 rotary and M3 Pro Gold NiTi files. Restorative Dentistry & Endodontics, 43(2), e25. 29765904 10.5395/rde.2018.43.e25 PMC5952062

[cre2893-bib-0020] Pruett, J. P. , Clement, D. J. , & Carnes, Jr. D. L. (1997). Cyclic fatigue testing of nickel–titanium endodontic instruments. Journal of Endodontics, 23(2), 77–85. 9220735 10.1016/S0099-2399(97)80250-6

[cre2893-bib-0021] Rashnooie, R. , Zeinoddini, M. , Ahmadpour, F. , Beheshti Aval, S. B. , & Chen, T. (2023). A coupled XFEM fatigue modelling of crack growth, delamination and bridging in FRP strengthened metallic plates. Engineering Fracture Mechanics, 279, 109017.

[cre2893-bib-0022] Recho, N. (2012). Fracture mechanics. In N. Recho (Ed.), Fracture mechanics and crack growth (pp. 87–186).

[cre2893-bib-0023] Scattina, A. , Alovisi, M. , Paolino, D. S. , Pasqualini, D. , Scotti, N. , Chiandussi, G. , & Berutti, E. (2015). Prediction of cyclic fatigue life of nickel–titanium rotary files by virtual modeling and finite elements analysis. Journal of Endodontics, 41, 1867–1870. 26361644 10.1016/j.joen.2015.07.010

[cre2893-bib-0024] Shahmoradi, M. , Wan, B. , Zhang, Z. , Swain, M. , & Li, Q. (2022). Mechanical failure of posterior teeth due to caries and occlusal wear—A modelling study. Journal of the Mechanical Behavior of Biomedical Materials, 125, 104942. 34800891 10.1016/j.jmbbm.2021.104942

[cre2893-bib-0025] Sjögren, U. , Hägglund, B. , Sundqvist, G. , & Wing, K. (1990). Factors affecting the long‐term results of endodontic treatment. Journal of Endodontics, 16(10), 498–504. 2084204 10.1016/S0099-2399(07)80180-4

[cre2893-bib-0026] Streeton, R. , Cooke, M. , & Campbell, J. (2004). Researching the researchers: Using a snowballing technique. Nursing Research, 12(1), 35–46. 10.7748/nr2004.07.12.1.35.c5929 15493213

[cre2893-bib-0027] Tian, R. , Wen, L. , & Wang, L. (2019). Three‐dimensional improved XFEM (IXFEM) for static crack problems. Computer Methods in Applied Mechanics and Engineering, 343, 339–367.

[cre2893-bib-0028] Valdivia, A. D. C. M. , Raposo, L. H. A. , Simamoto‐Júnior, P. C. , Novais, V. R. , & Soares, C. J. (2012). The effect of fiber post presence and restorative technique on the biomechanical behavior of endodontically treated maxillary incisors: An in vitro study. The Journal of Prosthetic Dentistry, 108(3), 147–157. 22944310 10.1016/S0022-3913(12)60138-3

[cre2893-bib-0029] Wan, B. , Entezari, A. , Zhang, Z. , Wilson, T. , Yoda, N. , Zheng, K. , Wu, C. , Sun, G. , Sasaki, K. , Swain, M. , & Li, Q. (2021). On fatigue failure prediction of prosthetic devices through XFEM analysis. International Journal of Fatigue, 147, 106160.

[cre2893-bib-0030] Wan, B. , Yoda, N. , Zheng, K. , Zhang, Z. , Wu, C. , Clark, J. , Sasaki, K. , Swain, M. , & Li, Q. (2022). On interaction between fatigue of reconstruction plate and time‐dependent bone remodeling. Journal of the Mechanical Behavior of Biomedical Materials, 136, 105483. 36302272 10.1016/j.jmbbm.2022.105483

[cre2893-bib-0031] Yahia, L. H. , Rayes, F. , & Warrak, A. O. (2009). Regulation, orthopedic, dental, endovascular and other applications of Ti–Ni shape memory alloys. In T. Yoneyama & S. Miyazaki (Eds.), Shape memory alloys for biomedical applications (pp. 306–326). Woodhead Publishing.

[cre2893-bib-0032] Yazid, A. , Abdelkader, N. , & Abdelmadjid, H. (2009). A state‐of‐the‐art review of the X‐FEM for computational fracture mechanics. Applied Mathematical Modelling, 33(12), 4269–4282.

[cre2893-bib-0033] Zhang, Y. , Guo, K. , & Jiang, S. (2022). Influence of amorphous phase on crack initiation and propagation behaviours in NiTi shape memory alloy based on extended finite element simulation. IOP Conference Series: Materials Science and Engineering, 1270(1), 012119.

[cre2893-bib-0034] Zhang, Y. , Liu, Y. , She, Y. , Liang, Y. , Xu, F. , & Fang, C. (2019). The effect of endodontic access cavities on fracture resistance of first maxillary molar using the extended finite element method. Journal of Endodontics, 45(3), 316–321. 30803539 10.1016/j.joen.2018.12.006

[cre2893-bib-0035] Zhang, Y. Y. , Peng, M. D. , Wang, Y. N. , & Li, Q. (2015). The effects of ferrule configuration on the anti‐fracture ability of fiber post‐restored teeth. Journal of Dentistry, 43(1), 117–125. 25456614 10.1016/j.jdent.2014.10.003

[cre2893-bib-0036] Zhou, L. , Wang, L. , & Jiang, L. (2022). Chapter 2—Materials of steel structures. In L. Zhou , L. Wang , & L. Jiang (Eds.), Design of steel structures (pp. 19–67). Elsevier.

[cre2893-bib-0037] Zhuang, Z. , Liu, Z. , Cheng, B. , & Liao, J. (2014). Chapter 1—Overview of extended finite element method. In Z. Zhuang , Z. Liu , B. Cheng , & J. Liao (Eds.), Extended finite element method (pp. 1–12). Academic Press.

[cre2893-bib-0038] Zupanc, J. , Vahdat‐Pajouh, N. , & Schäfer, E. (2018). New Thermomechanically treated NiTi alloys—A review. International Endodontic Journal, 51(10), 1088–1103. 29574784 10.1111/iej.12924

